# Design of a deep fusion model for early Parkinson’s disease prediction using handwritten image analysis

**DOI:** 10.1038/s41598-025-04807-6

**Published:** 2025-07-01

**Authors:** Shyamala K, Navamani T M

**Affiliations:** School of Computer Science and Engineering (SCOPE), Vellore Institute of Technology (VIT), Vellore, Tamil Nadu India

**Keywords:** Deep learning, Handwritten images, Parkinson’s disease, Disease prediction, Pre-trained CNN models, Neurological disorders, Neurodegenerative diseases, Parkinson's disease

## Abstract

Parkinson’s Disease (PD) is a deteriorating condition that mostly affects older people. The lack of conclusive treatment for PD makes diagnosis very challenging. However, using patterns like tremors for early diagnosis, handwriting analysis has become a useful diagnostic technique. This work aims to improve early PD diagnosis by proposing a hybrid deep fusion model that blends ResNet-50 and GoogLeNet (RGG-Net). We demonstrated the RGG-Net model in a series of steps such as preprocessing images, ResNet-50 and GoogLeNet models for feature extraction, combining the features using the Adaptive Feature Fusion technique and selecting the relevant features using the attention process, making the models stronger through Hierarchical Ensemble Learning. The grad-CAM technique is used for decision-making in PD prediction. The proposed model is a reliable way to analyze handwritten images using advanced techniques like adaptive feature fusion, hierarchical ensemble learning, and eXplainable Artificial Intelligence. Here, we analyzed ten pre-trained models to determine which model best captures the relevant features for PD classification using handwritten images. The models included are AlexNet, DenseNet-201, SqueezeNet1.1, VGG-16, VGG-19, ResNet-50, ResNet-101, GoogleNet, MobileNetV1, and MobileNetV2. The proposed deep transfer learning model showed an accuracy of 99.12%, outperforming the other state-of-the-art methods, indicating the model’s excellence and vigor. The proposed model performs better than all pre-trained models with and without freezing convolutional layers. These results underscore the efficacy of the proposed approach in enhancing accuracy and transparency in Parkinson’s disease prediction and the potential of deep learning in promoting early diagnosis.

## Introduction

Parkinson’s disease (PD) is a deterioration or degeneration of dopamine-producing nerve cells in the brain’s central nervous system that causes a loss of muscle function, sluggish movement, tremors, stiffness, and poor balance. PD is the second most prevalent degenerative disorder, next to Alzheimer’s disease. According to current research, India has 7 million older people affected with Parkinson’s disease. People in their middle and later years are more likely to be affected by Parkinson’s Disease. According to the Union Ministry and Family Healthcare Statistics, the average life expectancy of Indians has increased by 5 years in the previous decade. The United Nations Population Division (India) projects that this number will rise by 19% by 2050, which means that the illness burden in India is predicted to grow significantly. This will put tremendous pressure on the country’s already-straining healthcare system, which is already struggling to fulfill the needs of people from all walks of life and all corners of the realm^1^.

PD symptoms can be classified into motor and non-motor symptoms. In the first category, the patients suffer from symptoms including tremors, Freezing of Gait (FoG), Muscle rigidity, Fear of Falling (FoF), Slow Movements, Impaired Posture, Micrographia, and voice abnormality^2^. The symptoms of the second category consist of depression, dementia, sleep difficulties, anxiety, sluggish thinking, and exhaustion^3^. Parkinson’s disease is difficult to diagnose in advance of symptoms, and even when they do, it can be challenging to differentiate the condition from many other degenerative illnesses. However, it isn’t easy to achieve better accuracy. Changes in handwriting are frequently observed in Parkinson’s disease patients. When writing over extended periods, handwriting often gets tiny and clumsy and might become harder to control. Research suggests that handwriting tests showing Micrographia generally indicate PD^4^. Recently Machine Learning (ML) and Deep Learning (DL) have gained popularity in various sectors. ML and DL models are becoming more common in the healthcare sector due to technological advancement. ML and DL models extract essential data for PD prediction, which is necessary for the early stage of the disease and critical to disease prediction. Convolutional Neural Networks (CNNs) are popular deep learning techniques, especially for addressing the shortcomings of traditional machine learning methods^5,6^. The investigation of drawings has shown their value in Parkinson’s disease diagnosis. To enhance the patient’s life quality, digital approaches mostly rely on the precision of the model, thus it is critical to implement deep learning-based algorithms to obtain better accuracy for PD prediction and speed up the diagnostic process^7,8^. Convolutional Neural Networks (CNNs) have potential benefits in reducing the misdiagnosis rate of Parkinson’s disease. CNNs can learn difficult-to-see features, allowing them to identify PD symptoms that conventional diagnostic techniques might overlook. Additionally, CNNs can learn from large data points, including individuals with PD and other neurodegenerative disorders in the training collection, reducing the chance of mistakes^9^. Motivated by the catastrophic outcomes of misdiagnosis, our goal is to perform on different pre-trained models using the handwritten drawings dataset for early-stage Parkinson’s disease diagnosis.

Handwritten drawings are essential in predicting PD related to tremors(shaking). Examining a person’s ability to draw spirals and waves on a pre-made piece of paper is a common part of the handwriting assessment tool used to identify PD. Notable progress has been made in early-stage PD detection and available treatments in the last several years. Currently, PD is diagnosed through clinical evaluations, which is a laborious procedure that requires additional human specialists. Hence, PD prediction is an expensive and time-consuming process. As a result, automated early PD prediction is necessary in the medical field^10^. Even while handwriting alterations are rarely noticeable in the early stages of the disease, they are still a crucial indicator for diagnosing PD^11^. The CNN model automatically extracts visual information and uses that data to train a multilayer neural network to predict PD accurately. Better accuracy in image classification has been promised using DL^12,13^. CNNs are extensively employed in medical imaging because of their robustness in automated feature extraction, making them the most prevalent DL approach in this field^14–16^. On the other hand, when there is a shortage of data, it is better to use pre-trained models that have already been trained, like ImageNet, and “transfer” all the information in the model to be trained to the new data^17,18^.

The fundamental goal of this study is to improve the early detection of Parkinson’s Disease (PD) using deep learning methods, notably Convolutional Neural Networks (CNNs), and consider handwritten drawings as a diagnostic tool. Despite significant advances, early identification of Parkinson’s disease remains challenging owing to the difficulties separating its symptoms from those of other degenerative conditions. Current clinical examinations for Parkinson’s disease are time-consuming and sometimes need specialist knowledge, making early detection costly and labor-intensive. While considerable progress has been made in employing handwriting traits, such as micrographia, for diagnosis, current approaches are insufficiently accurate, especially in the early stages of the condition. Parkinson’s Disease (PD) prediction encounters several challenges in using handwriting alterations, such as Micrographia, for reliable and accurate PD diagnosis. Reliance on subjective evaluation in conventional diagnostic techniques results in possible misdiagnosis. We use deep learning techniques like Convolutional Neural Networks (CNNs) to lower the misdiagnosis and improve model accuracy to make better PD predictions. The limited dimensions of PD datasets exacerbate the issue, resulting in overfitting and reducing generalizability. Attaining an equilibrium between outstanding prediction efficacy and model interpretability is a considerable challenge since intricate systems sometimes lack transparency. The opacity of deep learning models, especially convolutional neural networks, undermines confidence and application in medical environments, complicating healthcare practitioners’ reliance on these technologies for clinical decision-making. This study attempts to fill these gaps by combining transfer learning and sophisticated feature fusion approaches to increase the accuracy of automated PD prediction models. The motivation derives from the rising burden of PD in India and the globe and the need for practical, automated methods to minimize misdiagnosis rates and speed up the diagnostic process, thereby improving patient outcomes.

In^19^, the authors provided a method to improve the identification of Parkinson’s disease by merging several handwritten datasets and utilizing advanced deep-learning algorithms. Their approach involves data augmentation, extracting features using pre-trained Convolutional Neural Networks (CNNs), and merging the extracted features. The authors emphasize the need to investigate more sophisticated fusion tactics, attention processes, and explainable AI techniques to enhance the model’s performance and comprehensibility. In^20^, the authors proposed a novel approach for PD diagnosis using a deep neural network and applied the Harris Hawks Optimization algorithm. The authors utilized a handwritten dataset and achieved an impressive accuracy of 94.12%. It highlights the potential of deep learning for PD diagnosis, emphasizing the need for larger, more diverse datasets and continuous monitoring of patient handwriting for enhanced diagnostic accuracy. Hence, we propose a hybrid transfer learning fusion model to predict early Parkinson’s disease (PD) using a handwritten dataset to address the mentioned problems.

The main contributions of this study are as follows.

- Proposed a hybrid deep fusion model for early PD prediction to improve classification accuracy.
- Assessing the prediction of early Parkinson’s disease using handwritten images with pre-trained convolutional neural network architectures and convolutional layer freezing.
- Exploring the impact of CNN architecture variants and hyperparameter tuning on transfer learning performance for PD prediction.
- Extensive comparative analysis of existing techniques with the proposed model for early PD diagnosis using handwritten images is done.

The remaining structure of the work is outlined as follows. “Related works” analyzes the related works on PD prediction. “Methodology” involves the dataset description, proposed RGG-Net model, pre-trained models, and performance metrics. “Results and discussion” focuses on the results of experimental outcomes, comparative assessment, and the incorporation of eXplainable Artificial Intelligence (XAI). Finally, “Conclusion” summarizes the work and pinpoints the future research directions toward PD prediction.

## Related works

Artificial intelligence (AI) in healthcare is becoming increasingly popular in this rapidly evolving era. Early diagnosis of Parkinson’s Disease (PD) has been the subject of extensive research throughout the years. For instance, handwriting has emerged as a potentially useful biomarker for early Parkinson’s disease diagnosis in recent years. This is because handwriting is used to evaluate a person’s cognitive and motor abilities, and handwriting’s graphical features can identify the ambiguity of strokes caused by shaking movements.

Variations in the printed character size, text block region, height of loop patterns, pixel thickness variances resulting from ink content, density, height ratios, and curved accuracy index are examples of predictable metrics^21,22^. These investigations help assess the severity of PD and make it easier to track the disease’s course over the period and identify early signs. For instance, a study looked at the viability of using passive analysis to assess the severity of PD in ten patients by assessing handwritten sample images based on handwriting history. Many ML techniques are used to distinguish between PD and healthy control. The authors of the article^23^ used SVM in several handwriting tasks and kinematic and pressure parameters to achieve an accuracy rate of 81.3%, which includes 37 Parkinson’s Disease affected roles and 38 healthy controls.

The authors^24^ have compared state-of-the-art deep learning techniques using the Hand Parkinson’s Disease dataset. CNN was used to distinguish between handwritten sample images, comprising 18 healthy controls and 74 people with Parkinson’s disease with distinct features. In^25^, the authors used different prediction methods for PD classification and showed that neural networks are better at classification than other classifiers with 92.2% accuracy. The authors of reference^26^ proposed a model to increase the accuracy of automated PD diagnosis across the handwritten PD dataset by utilizing several classifiers. The findings showed that the ANN classifier method works better than the Linear Regression (LR) and Random Forest (RF) classifiers. Using a dynamic handwritten dataset, the authors^27^ used deep Optimum-Path Forest (OPF) clustering for PD prediction. Subsequently, In^28^, the authors reported a PD classification model based on a standardized variable velocity of the handwritten dataset. The balanced dataset was used in the work.

The authors in^4^ created a CNN to separate the relevant features of the handwritten images for PD and the classification model has a 96.5% accuracy rate. The authors^29^ used the PaHaW dataset to apply transfer learning via AlexNet to discriminate PD from healthy individuals. The authors used optimized ImageNet features to reach a 98.28% classification accuracy. The study^30^ noted that digitalized spiral hand drawings are a viable input for PD classification. They summarized findings with a precision of 91% and an AUC of 98.1% using four machine-learning classifiers that were applied to the feature-engineered dataset after it had been mathematically processed. Histogram of Oriented Gradients (HOG) feature descriptors used to predict early stage PD patients using hand-drawn wave and spiral images. Several machine learning classifier methods are suggested, and KNN performs better in^31^. The authors of the article^32^ used Alex Net and GoogLeNet models to analyze the performance of PD using dynamic spiral drawings, and the precision rate is 94%. The study^33^ investigated spiral pentagon drawings using the convolutional layer to distinguish PD patients from non-PD and yielded a precision of 93.5%.

The study^34^ developed a multi-modal diagnosis system that integrates EEG signals from the Substantia Nigra (SN) and Ventral Tegmental Area (VTA) with handwriting tasks. The system employs Grey Wolf Optimization (GWO) for feature selection and Bi-directional Long Short-Term Memory (BLSTM) for classification. The system achieved an impressive accuracy of 99.30% on the ESOH dataset. Nevertheless, the complexity of real-world data acquisition and the restricted dataset size present obstacles to broader applicability. In^35^, the authors developed a transfer learning-based model that optimized feature selection using genetic algorithms and K-Nearest Neighbors (KNN). This model achieved over 95% accuracy and exhibited superior detection capabilities compared to state-of-the-art methods. However, the model’s capacity for multimodal analysis and custom architecture exploration is restricted by its dependence on handwriting data and pre-trained networks. In contrast, the study^36^ utilized deep learning algorithms to identify motor impairments through spiral and wave patterns, applying Convolutional Neural Networks (CNNs) and ensemble classifiers to handwriting tasks. This approach yielded competitive results. Nevertheless, the clinical relevance of this study is limited by its restricted focus on handwriting data and the absence of multimodal integration. Collectively, these studies emphasize the potential of integrating sophisticated optimization techniques, multimodal datasets, and explainable AI to improve diagnostics’ accuracy and real-world applicability in Parkinson’s disease research. In^37^, the authors highlighted the ease and scalability of Convolutional Neural Networks (CNNs) in therapeutic contexts, proposing its use to extract features from spiral and sinusoidal drawings. Using features extracted from Histograms of Oriented Gradients (HOG), the method achieved a classification accuracy of 83.1%. However, research into multimodal data amalgamation is limited due to the reliance on traditional drawing tasks. Transfer learning and more hyperparameter tuning are needed to improve accuracy. According to^38^, PD can be better detected by integrating speech and handwriting analysis. Better accuracy can be achieved in the hybrid model than in the single-modality models when the handwriting and voice data are processed using CNNs. However, issues with feature fusion and dataset diversity were discovered.

Several notable works have been examined in the literature review to contextualize the current research. The authors of^39^ conducted a study utilizing 204 spiral and wave PD drawings, employing DenseNet201 and VGG-16 models. Their findings demonstrated 94% accuracy with DenseNet-201 on spiral images and 90% accuracy with VGG-16 on wave images. However, this study was limited to evaluating only two pre-trained models, neglecting the potential benefits of exploring a wider range of models. Similarly, The authors of reference^40^ investigated two datasets comprising 102 spiral and wave drawings and 265 spiral drawings, respectively, employing VGG-19 and GoogLeNet. Their hybrid transfer learning model achieved an accuracy of 98.45%, emphasizing employing explainable AI for decision-making. Nevertheless, the study overlooked data imbalance issues and suggested combining multiple pre-trained models to enhance PD prediction and severity assessment. The study^41^ analyzed 102 spiral and wave drawings from a handwriting dataset, utilizing the VGG-19 model and achieving 88% accuracy for waves and 89% for spirals. However, their study solely relied on VGG-19 without exploring alternative pre-trained models for evaluation. The authors^42^ have examined a handwriting dataset employing Random Forest, CNN, and ResNet-50 models, reporting varied accuracies across models, with Random Forest producing lower accuracy than ResNet-50. Despite this, the study recommended the inclusion of comparisons with other variants of CNN pre-trained models for comprehensive evaluation. Finally, The study^43^ employed a simple CNN model on a handwriting dataset, achieving an accuracy of 63% for PD prediction. However, the study’s reliance on a basic CNN model underscores the potential benefits of exploring more complex architectures for improved accuracy. Existing research showcases the promise of DL for early PD diagnosis. However, it also highlights the limitations of using a single pre-trained model, the failure to address data imbalance, and the potential for improvement through exploring different CNN architectures and combining models. Thus, we aim to address these gaps by investigating the efficacy of various pre-trained CNN architectures with convolutional layer freezing for early PD diagnosis.

Recent research shows that pre-trained models are required to improve PD performance and address overfitting and computational resources. Additionally, there is a need to explore the existing pre-trained models for PD prediction. Also, it is observed from the literature that comprehensive pre-trained models, comparative analysis, and the investigation of the impact of convolutional layers on PD prediction are not adequately done. We planned to propose a hybrid pre-trained fusion model for PD prediction to overcome the mentioned issues. Also, we consider ten CNN variant models with and without freezing convolutional layers for evaluation analysis. In conclusion, these studies solidify the potential of handwriting analysis, particularly when combined with deep learning techniques for PD prediction and monitoring. Notably, DL methods, specifically Convolutional Neural Networks (CNNs), have emerged as particularly successful tools, providing better performance in disease diagnosis. Additionally, pre-trained models are adapted for PD diagnosis and have proven effective in medical analysis. This study offers significant promise for early PD diagnosis, monitoring disease progression, and potentially improving patient care.

## Methodology

This section covers the dataset description, deep transfer learning, the proposed RGG-Net model, pre-trained models, and performance metrics. The proposed RGG-Net model is presented with a sequence of processes such as image preprocessing, ResNet-50 and GoogLeNet models for feature extraction, Adaptive Feature Fusion and Hierarchical Ensemble Learning, Classification, and eXplainable Artificial Intelligence.

### Dataset description

This module provides an extensive overview of the dataset, preprocessing, and augmentation steps. K Scott Mader created a collection of datasets for both healthy control and Parkinson’s disease individuals which is available on Kaggle^44^. The set of handwritten drawings has 204 images in total. Nevertheless, the dataset was insufficient to train the model for improved classification because of the limited data available. A common problem in deep learning applications is a shortage of data, which can significantly limit the model’s performance. To get around this problem, data augmentation has been used to expand the size of the training dataset artificially. Random translations, scaling, reversals, rotations, and other operations that maintain the original image’s label are all included in data augmentation. The data is enhanced in this work by turning the data 180°vertically, rotating 90°, 180°, and 270°, and then all of it is turned to color images^45^.

**Fig. 1:**
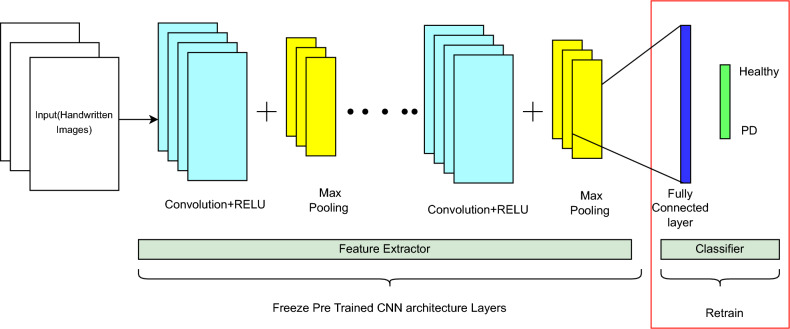
A typical pre-trained CNN architecture.

A total of 3264 original images, including 1632 healthy control and 1632 Parkinson’s disease-related images obtained after augmentation, are used to assess the effectiveness of hybrid deep transfer learning. Pre-trained models from AlexNet, Dense Net 201, SqueezeNet1.1, VGG-16, VGG-19, ResNet-50, ResNet-101, MobileNetV1, and MobileNetV2 were employed in this work. However, these models only functioned with 224 x 224 image resolution. The difference between the necessary and gathered image pixels is significant. Thus, the resizing process is done by normalizing pixel data to the range [0,1]. The entire dataset is divided into 70%, 20%, and 10% for training, testing, and validation. Following that, the outcomes of the preprocessing step will be inputs for the hybrid deep transfer learning model.

### Transfer learning

Transfer learning (TL) uses knowledge acquired from pre-trained models and enhances it by fine-tuning it with domain-specific data^46^. Although training the model from scratch by assigning random weights is an option, setting the pre-trained model weights is more advantageous to improve the network’s output on large public datasets^47,48^. Transfer learning identifies appropriate networks and assigns the weights of models to the lower layers of the CNN^49^. Figure 1 depicts the pre-trained CNN architecture for PD prediction, comprising convolutional, ReLU, Max Pooling layers, and a fully connected layer. PD impairs a person’s motor abilities, frequently leading to observable alterations in handwriting like tremors and diminished amplitude. Figure 1 depicts the pre-trained CNN model that utilizes a feature extraction phase with convolution and pooling layers to analyze handwritten images. The model can accurately detect the existence of PD based on handwritten image analysis by retraining the classifier layer using labeled data showing both healthy control and PD-affected handwriting.

**Fig. 2:**
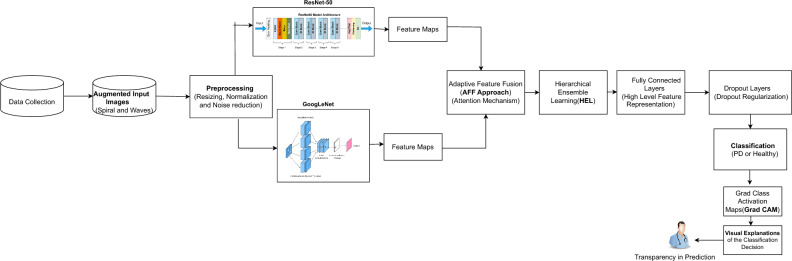
Proposed architecture of RGG-Net model.

### Proposed architecture of RGG-Net

Figure 2 outlines a comprehensive workflow for predicting PD using handwritten images, incorporating Deep Learning techniques and Gradient-weighted Class Activation Maps (Grad-CAM) for visual interpretation. This research study focused on evaluating the performance of the hybrid deep transfer learning model. The architecture of the ResNet-50 and GoogLeNet (RGG-Net) fusion model is illustrated in Fig. 2. The proposed approach utilizes the ResNet-50 and GoogLeNet models to create an enhanced transfer learning fusion model for Parkinson’s disease prediction. The integration of ResNet-50 and GoogLeNet involves merging the extracted features from both networks to capture a richer representation of the handwritten images. This combined feature representation enables the model to discern intricate patterns and subtle variations crucial for accurate PD classification. By freezing the layers of both models during training, the architecture preserves the learned features while allowing the custom classifier to adapt to the relevant features of the PD dataset.

This architecture combines the strengths of ResNet-50 deep residual learning and GoogLeNet’s inception modules for more accurate and robust feature extraction and classification. The ResNet-50 network captures fundamental elements from handwritten images, whereas the Inception model captures high-dimensional data. Using pre-trained models is more advantageous than conventional ML models, without pre-trained models or weights, or training the model entirely with a limited dataset to differentiate between PD and non-PD, as it helps mitigate overfitting. Algorithm 1 showed the steps involved in the hybrid deep transfer learning model.

**Algorithm 1:**
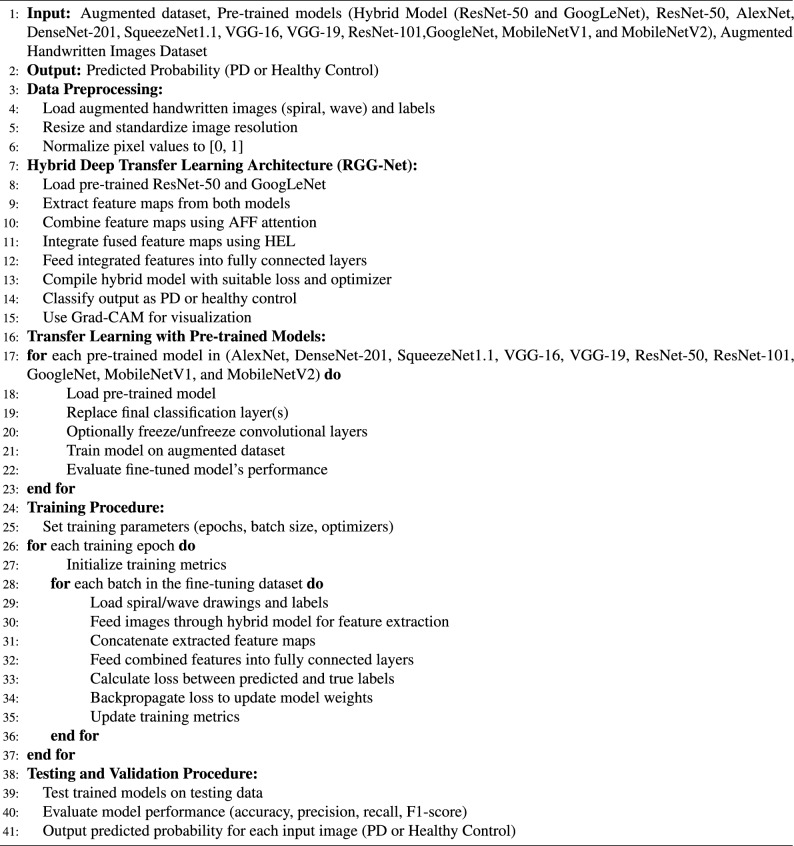
Pseudocode-Comprehensive Pre-trained CNN Models using Handwritten Images for PD Prediction

We have taken input images as augmented images to increase the diversity of the dataset and strengthen the model’s capacity to handle different scenarios. Subsequently, the enhanced images undergo preprocessing procedures, including scaling, normalization, and noise reduction, to prepare them for feature extraction. For feature extraction, the preprocessed images are fed into two pre-trained Convolutional Neural Networks (CNNs) on ImageNet: ResNet-50 and GoogLeNet. Each network extracts appropriate feature maps. The feature maps derived from these models are subsequently merged using an Adaptive Feature Fusion (AFF) technique that utilizes attention processes to concentrate on the most relevant features. The combined features are analyzed using a Hierarchical Ensemble Learning (HEL) framework to capitalize on the strengths of the two models and provide a sophisticated feature representation. The Adaptive Feature Fusion (AFF) approach aims to optimally integrate the features retrieved by the two pre-trained models, ResNet-50 and GoogLeNet. The AFF method ensures that the fusion process is adaptive, so the model learns which features from every pre-trained model are most crucial for prediction making. Although extracting features from handwritten images, the ResNet-50 and GoogLeNet models extract features from different types or levels of abstraction. An adaptive procedure aggregates the features taken from both models. In this situation, the AFF uses attention processes to evaluate the relevance of every component. This guarantees that the model focuses on the essential features to differentiate PD from Healthy Control (HC). The attention mechanism assigns additional weights to more information elements, allowing the model to focus on some visual regions or patterns showing PD. This helps in filtering out irrelevant or noisy information. The fused features help the model to correctly recognize handwritten images and better reflect the intricate patterns in the data. Hierarchical Ensemble Learning (HEL) is a method for layered, orderly combining many models or features. In RGG-Net, HEL uses the outputs of the ResNet-50 and GoogLeNet models after the fusion procedure to provide a more complex and intense classification choice. Combining predictions from many models is the essence of ensemble learning to raise the general performance level. ResNet-50 and GoogLeNet’s feature maps are processed via an ensemble procedure in HEL, where they are examined jointly, considering their hierarchical linkages. HEL’s hierarchy indicates that the model arranges the features in layers, so higher-level features (more abstract representations) are merged first, then the lower-level features, rather than directly combining the features. Continuously improving its grasp of the data, this approach helps the model to make more correct judgments. The handwritten image representation must be improved for better classification results from the hierarchical fusion of features. It uses the complementary strengths of the two networks, ResNet-50 for basic features and GoogLeNet for high-dimensional features, to provide a more significant total feature representation.

Subsequently, this representation is sent via fully connected layers to undergo additional processing and abstraction. At this stage, dropout regularization is implemented to mitigate the risk of overfitting. Ultimately, the analyzed features are categorized as either Parkinson’s Disease or Healthy Control. Grad-CAM (Gradient-weighted Class Activation Mapping) is utilized to construct activation maps, visually illustrating the key regions in the images that have affected the classification decisions. Algorithm 1 utilizes a hybrid deep transfer learning architecture integrating ResNet-50 and GoogLeNet models to boost the accuracy of PD prediction utilizing augmented handwriting images. At first, the augmented dataset is preprocessed by scaling and normalizing the spiral and wave images that make up the dataset. The feature maps employed by the hybrid model are extracted using RGG-Net to extract important features and are merged using the attention mechanism. Before being classified as PD or healthy control, these fused features undergo high-level representation through fully linked layers. The Grad-CAM technique utilizes important features of the images for visualization. Also, we considered ten pre-trained models such as ResNet-101, ResNet-50, MobileNetV1, MobileNetV2, AlexNet, DenseNet-201, SqueezeNet1.1, VGG-16, VGG-19, and GoogleNet which are mentioned in the algorithm 1. The models are fine-tuned to make sure the predictions are solid by changing out the last classification layers and freezing or unfreezing the convolutional layers. The training method involves setting the parameters followed by iterative training and evaluation to refine the models. Finally, performance metrics are evaluated for PD classification. This comprehensive approach ensures accurate and interpretable predictions for Parkinson’s disease. The hybrid approach offers a complete and robust solution for medical image analysis by providing model interpretability using Grad-CAM and incorporating sophisticated techniques like adaptive feature fusion and hierarchical ensemble learning.

**Table 1:** CNN parameters used for different pre-trained models.

Architecture	Layer	Parameters
AlexNet	First convolutional layer	11 $\times$ 11 kernel size, 96 filters
	Second convolutional layer	5 $\times$ 5 kernel size, 256 filters
	Third convolutional layer	3 $\times$ 3 kernel size, 384 filters
	Fourth convolutional layer	3 $\times$ 3 kernel size, 384 filters
	Fifth convolutional layer	3 $\times$ 3 kernel size, 256 filters
DenseNet-201	Dense block layers	1 $\times$ 1 and 3 $\times$ 3 convolutional layers in dense blocks (201 layers)
SqueezeNet1.1	Fire modules	1 $\times$ 1 and 3 $\times$ 3 convolutional layers in fire modules
VGG-16	First convolutional layer	3 $\times$ 3 kernel size, 64 filters (twice)
	Second convolutional layer	3 $\times$ 3 kernel size, 128 filters (twice)
	Third convolutional layer	3 $\times$ 3 kernel size, 256 filters (three times)
	Fourth convolutional layer	3 $\times$ 3 kernel size, 512 filters (three times)
VGG-19	First convolutional layer	3 $\times$ 3 kernel size, 64 filters (twice)
	Second convolutional layer	3 $\times$ 3 kernel size, 128 filters (twice)
	Third convolutional layer	3 $\times$ 3 kernel size, 256 filters (three times)
	Fourth convolutional layer	3 $\times$ 3 kernel size, 512 filters (four times)
ResNet-50	Residual blocks	1 $\times$ 1, 3 $\times$ 3, and 1 $\times$ 1 convolutional layers in residual blocks; skip connections (50 layers)
ResNet-101	Residual blocks	1 $\times$ 1, 3 $\times$ 3, and 1 $\times$ 1 convolutional layers in residual blocks; skip connections (101 layers)
GoogleNet	Inception modules	1 $\times$ 1, 3 $\times$ 3, and 5 $\times$ 5 convolutions with max pooling; 22 layers
MobileNetV1	Depth-wise separable convolutions	3 $\times$ 3 depth-wise convolutions followed by 1 $\times$ 1 pointwise convolutions (28 layers)
MobileNetV2	Depth-wise separable convolutions with bottlenecks	3 $\times$ 3 depth-wise convolutions followed by 1 $\times$ 1 pointwise convolutions with linear bottlenecks (53 layers)

**Table 2:** Configuration details for the hyperparameters.

Hyperparameter	Value
Learning rate strategy	Adaptive LR scheduler (reduce on plateau)
Learning rate value	0.0001
Batch size	32
Optimizer	Adam
Loss function	Cross-entropy loss
Epochs	50
Frozen vs. unfrozen	Explicitly highlighted in results for consistency with frozen/unfrozen configurations

**Fig. 3:**
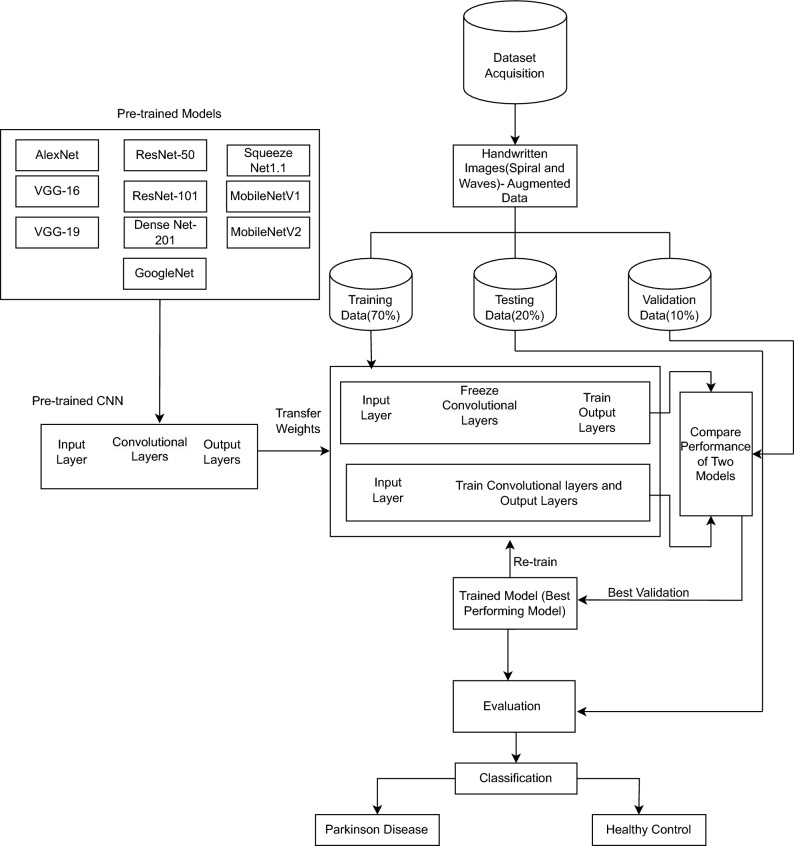
Frozen and unfrozen convolutional layers in pre-trained CNN models.

Table 2 summarizes the hyperparameters and their values tuned used to train the models in this study. The learning rate approach was an Adaptive Learning Rate Scheduler (Reduce on Plateau), which dynamically changes the learning rate during training to promote convergence. Depending on the model, the learning rate value is fixed as 0.0001 to ensure optimum performance. A batch size of 32 was used consistently across all models, combining computational efficiency with training stability. The Adam optimizer was selected because it effectively reduced the Cross-Entropy Loss, which served as the loss function for classification tasks. The models were trained for 50 epochs, which is adequate to achieve convergence without overfitting. Furthermore, the findings specifically compare frozen and unfrozen configurations of convolutional layers, which is consistent with the comparative analysis reported in the work.

#### Pre-trained models with and without freezing convolutional layers for Parkinson’s disease prediction

Pre-trained models are commonly employed as the foundation for making predictions in deep learning. Transfer Learning is the application of previously learned skills and information to solve novel problems or gain new knowledge in unfamiliar contexts. Freezing convolutional layers speeds up training and minimizes overfitting, while fine-tuning might increase accuracy.

Transfer learning enables the rapid retraining of a model without the need to retrain the entire model. Consequently, certain initial weights are immobilized, while others are employed to compute loss and are modified anytime the optimizer executes. The outcomes include accelerated training durations and reduced resource consumption, but perhaps at the expense of the ultimate accuracy of the trained model. The baseline pre-trained models are AlexNet^50^, DenseNet-201^51^, SqueezeNet1.1^52^, VGG-16^53^, VGG-19^53^, ResNet-50^54^, ResNet-101^54^, MobileNetV1^55^, and MobileNetV2^56^ were used in the study. This study utilizes the convolutional layer structure of pre-trained models, as depicted in Table 1. Figure 3 illustrates the models in both frozen and unfrozen phases.

The methodology developed for classifying PD employs a comprehensive approach with pre-trained CNN models. The initial phases of the process entail obtaining and processing a collection of handwritten spiral and wave images, which is essential for enhancing the dataset. The expanded dataset is subsequently split into training (70%), testing (20%), and validation (10%) subsets to aid in the model development and assessment of the model. Various pre-trained Convolutional Neural Network (CNN) models are used for PD prediction, as shown in Fig. 3. Each model undergoes specific transfer learning techniques, such as freezing the convolutional layers to retain the acquired features. Models are adjusted by repeated training, validation, and evaluation of the performance measures. The optimum configuration is then picked for final testing, which is done using an independent testing dataset. This systematic procedure is designed to improve the precision and dependability of PD classification, resulting in a classification conclusion that distinguishes between Parkinson’s disease and healthy control cases. In summary, the proposed approach presents a deep fusion model that combines ResNet-50 and GoogLeNet to diagnose PD. The model achieves an accuracy of 99.12% and demonstrated greater performance than the other ten pre-trained models. The results highlight the effectiveness of DL in enhancing the accuracy and transparency of Parkinson’s disease prediction. Thus, the proposed approach reveals the potential of deep learning to develop early indicative methods in medical imaging.

### Performance metrics

Metrics are used to measure performance and aid in evaluating and evaluating the effectiveness of the model. In general, metrics help to contrast the other classification models and identify the optimal model with respect to each parameter. The confusion matrix assesses the accuracy of DL models’ predictions, distinguishing between accurate and erroneous classifications. It has four categories: True Positive (TP), True Negative (TN), False Positive (FP), and False Negative (FN).

1 $$\begin{aligned} & \text {Accuracy} = \frac{TP + TN}{TP + TN + FP + FN} \end{aligned}$$

2 $$\begin{aligned} & \quad \text {Precision} = \frac{TP}{TP + FP} \end{aligned}$$

3 $$\begin{aligned} & \quad \text {Recall} = \frac{TP}{TP + FN} \end{aligned}$$

4 $$\begin{aligned} & \quad \text {F1 Score} = 2 \cdot \frac{\text {Precision} \cdot \text {Recall}}{\text {Precision} + \text {Recall}} \end{aligned}$$

5 $$\begin{aligned} & \quad \text {AUC} = \frac{1}{2} \left( \frac{TP}{TP + FN} + \frac{TN}{FP + TN} \right) \end{aligned}$$

6 $$\begin{aligned} & \quad \text {MCC} = \frac{(TP \cdot TN) - (FP \cdot FN)}{\sqrt{(TP + FP)(TP + FN)(TN + FP)(TN + FN)}} \end{aligned}$$

**Table 3:** Comparative analysis of pre-trained CNN models with the proposed RGG-Net model.

Model	Accuracy (frozen)	Accuracy (unfrozen)	Error rate	Training loss	Validation loss
AlexNet	93.39	92.0	20.3	0.3999	0.4174
DenseNet-201	97.2	96.8	10.4	0.3025	0.2249
SqueezeNet1.1	91.32	90.9	5.5	0.3254	0.1960
VGG-16	90.97	89.2	9.3	0.2012	0.3012
VGG-19	96.5	94.0	9.4	0.2116	0.3145
ResNet-50	96.09	95.5	4.1	0.4764	0.3483
ResNet-101	96.65	95.9	3.9	0.3756	0.3223
MobileNetV1	97.46	95.1	5.9	0.2234	0.3045
MobileNetV2	98.23	96.0	5.2	0.2198	0.2945
GoogleNet	95.01	94.43	4.2	0.4954	0.4573
Proposed model (RGG-Net)	99.12	96.5	1.6	0.0112	0.0183

**Fig. 4:**
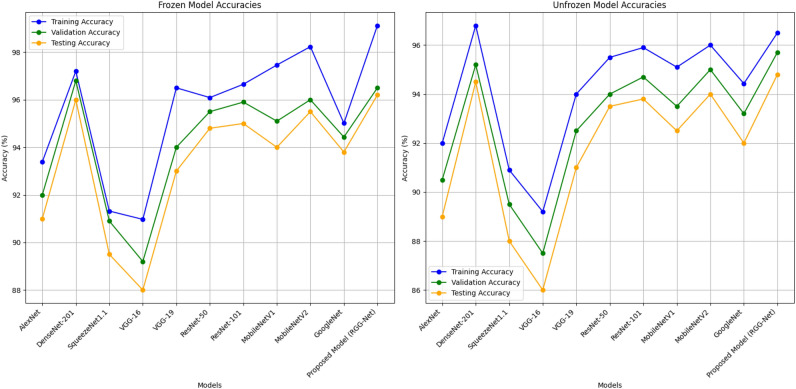
Pre-trained models with or without frozen convolutional layers based on training, validation, and testing accuracies.

**Fig. 5:**
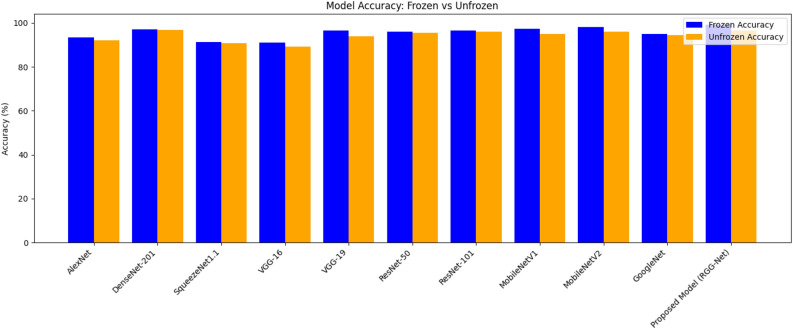
Comparison of model accuracies with frozen and unfrozen convolutional layers across different pre-trained models and proposed model.

Figure 5 compares the accuracy of pre-trained models developed using convolutional layers that were either frozen or not frozen. When the layers are frozen, the accuracy is blue, but when they are not, it is orange. With a maximum accuracy of 99.12% in the frozen scenario and 96.5% in the unfrozen scenario, the proposed model (RGG-Net) has the superior architecture. DenseNet-201 and MobileNetV2 perform similarly well in both cases, with minimal changes between frozen and unfrozen layers. Models such as AlexNet and VGG-16 have somewhat lower accuracies, suggesting that specialized training procedures, such as layer freezing or unfreezing, may be more effective. The accuracy difference between frozen and unfrozen layers is modest for most models, demonstrating that both techniques may provide good performance depending on the design. This comparison sheds light on how freezing convolutional layers might influence different neural network models’ learning ability and generalization.

**Table 4:** Comparative analysis of pre-trained models with frozen convolutional layers.

Model	Precision	Recall	F1-measure	MCC
AlexNet	92.46	92.42	92.39	91.92
DenseNet 201	96.85	96.80	96.82	96.60
SqueezeNet1.1	90.32	90.34	90.22	89.87
VGG-16 Net	90.08	89.82	89.77	89.17
VGG-19 Net	90.85	90.80	90.82	90.60
ResNet-50	95.09	94.91	94.93	94.58
ResNet-101	95.75	95.39	95.43	95.10
MobileNetV1	96.56	96.24	96.45	96.34
MobileNetV2	97.56	97.34	97.45	97.10
GoogleNet	94.68	94.01	94.53	94

**Fig. 6:**
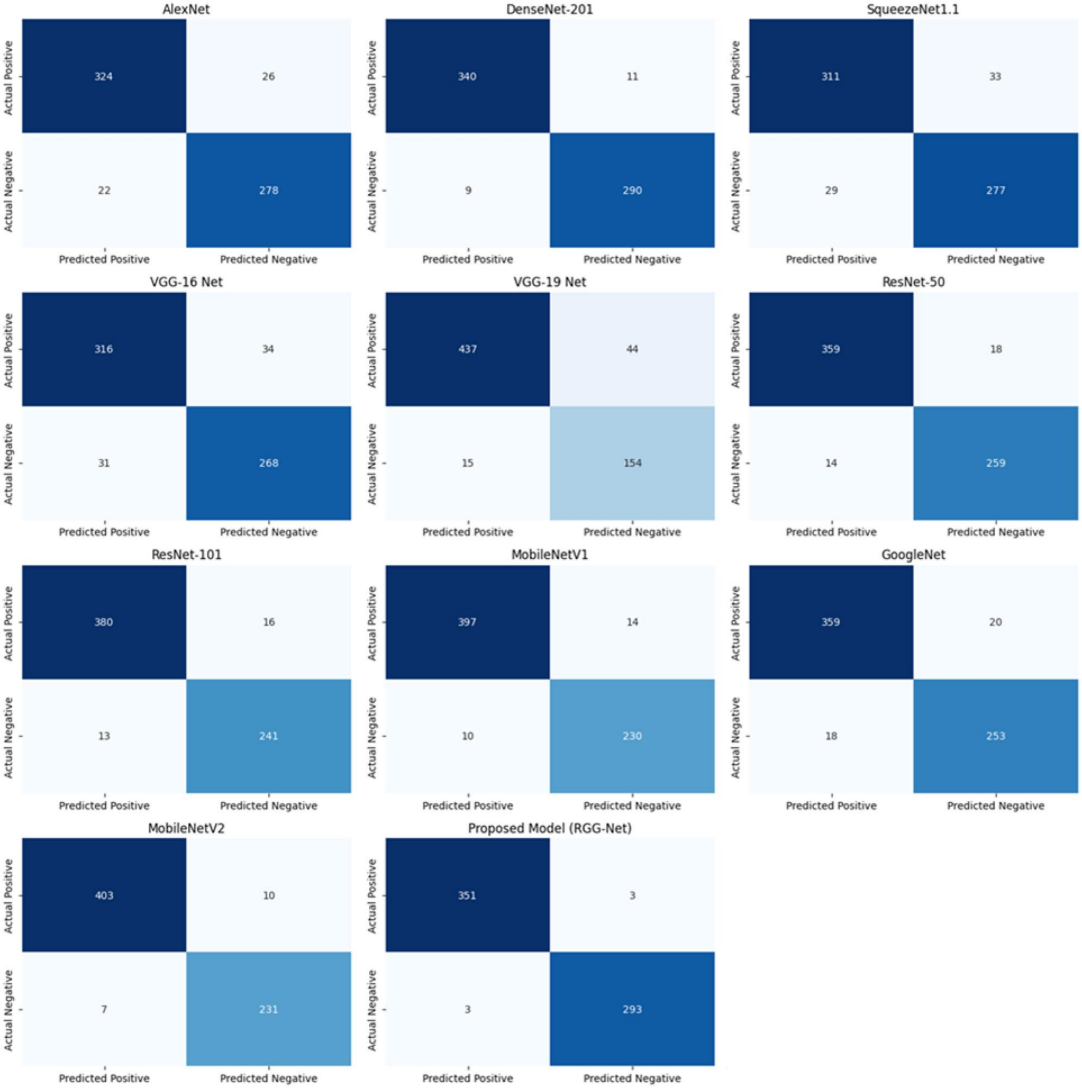
Confusion matrix for pre trained models with freezing.

**Table 5:** Comparative analysis of pre-trained models without frozen convolutional layers.

Model	Precision	Recall	F1-measure	MCC
AlexNet	91.1	91.0	90.8	90.5
DenseNet 201	96.5	96.2	96.3	96.1
SqueezeNet1.1	90.2	90.2	90.1	89.6
VGG-16 Net	88.2	88.0	87.9	87.3
VGG-19 Net	89.6	89.4	89.5	89.0
ResNet-50	94.4	94.1	94.2	93.8
ResNet-101	95.3	95.0	95.1	94.7
MobileNetV1	96.8	96.5	96.6	96.4
MobileNetV2	97.4	97.1	97.2	97.0
GoogleNet	92.21	94.67	94.67	94

**Fig. 7:**
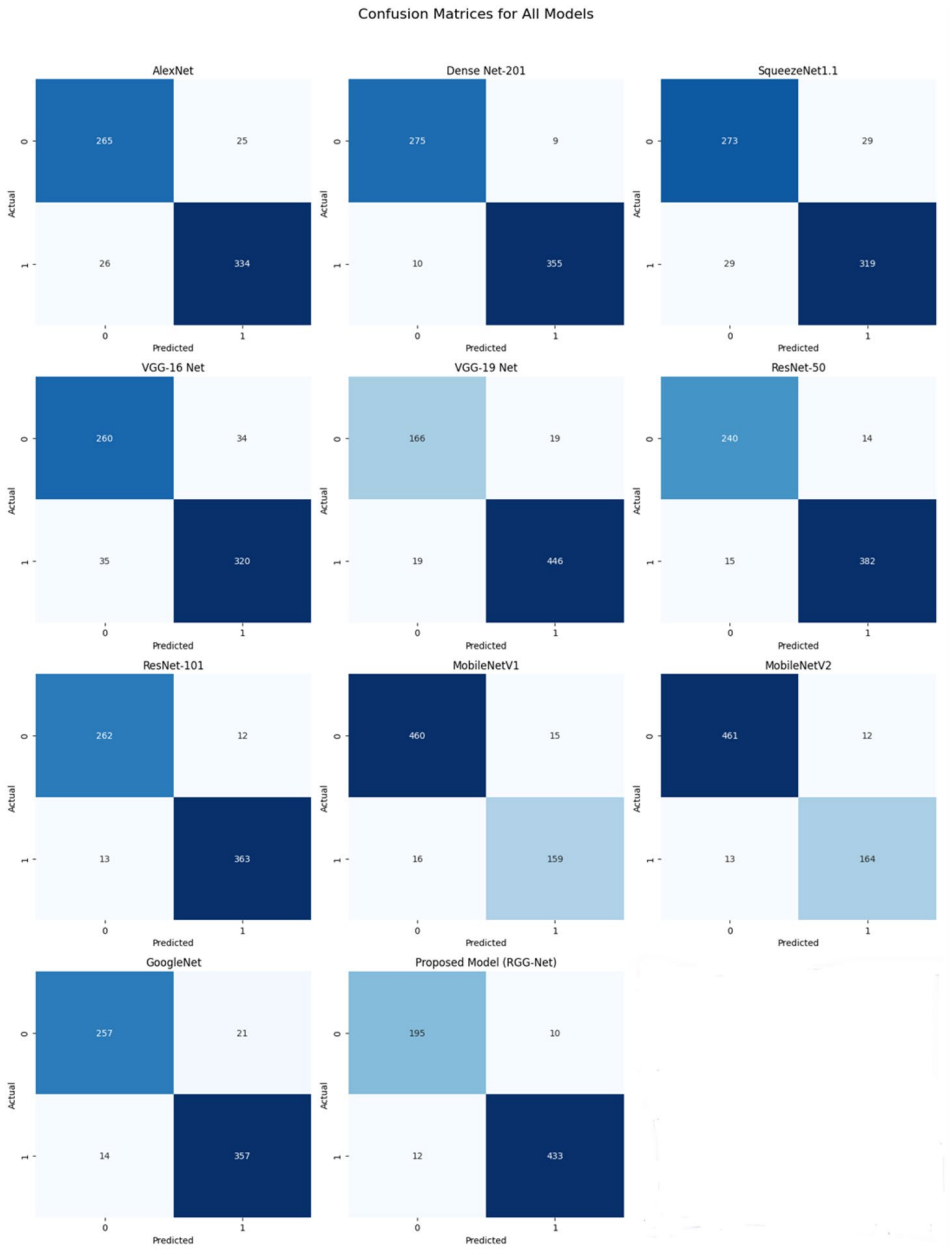
Confusion matrix for pre-trained models without freezing.

Accuracy, Recall, F1-measure, Precision^57^, and Matthews Correlation Coefficient (MCC) were used to evaluate the outcome of pre-trained models. MCC is a statistical tool used for model evaluation to differentiate between predicted and actual values. The result analysis section uses performance metrics to evaluate the performance metrics of PD prediction.

**Fig. 8:**
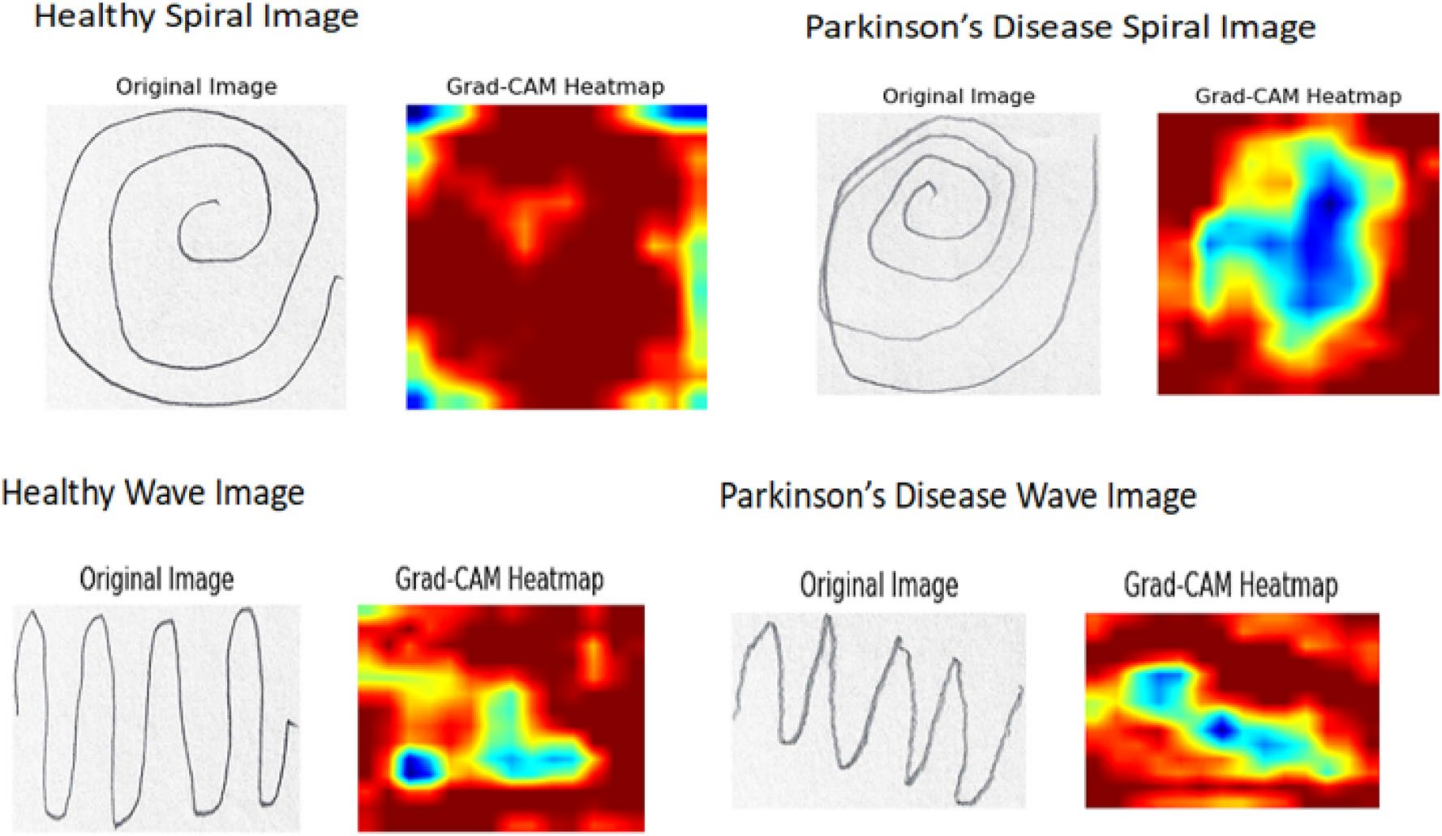
Activation maps for a handwritten dataset using the Grad-CAM technique.

## Results and discussion

This section comprehensively describes the exploratory setup, evaluation of performance criteria, and results for the DL models employed in the PD prediction task. In addition, we discuss the results of the Grad CAM technique used. The experimental configuration illustrates the efficacy of using pre-trained CNN architectures and a bespoke feature fusion model (RG-GNet) to classify Parkinson’s Disease (PD). Integrating an Intel Core i7 CPU with Google Colab’s T4 GPU facilitated the effective management of computationally demanding activities, ensuring prompt test execution.

This section presents the results of the PD prediction utilizing ten pre-trained models with and without freezing convolutional layers. The diagnostic precision of the models, both freezing and unfreezing convolutional layers, is presented in Table 3 and displays a comparative evaluation of different pre-trained models for Parkinson’s disease prediction. Models often exhibit greater accuracy when the convolutional layers are kept frozen rather than unfrozen. This might be attributed to the fact that the pre-trained features exhibited a high level of effectiveness, and adjusting the completely linked layers is adequate for accomplishing the prediction. The proposed model gets a better accuracy of 99.12% when the convolutional layers are frozen. This model greatly outperforms the other models. In summary, Table 3 provides evidence for the effectiveness of transfer learning in medical image processing tasks. It suggests that fine-tuning fully connected layers while leaving convolutional layers fixed can yield impressive accuracy results. We calculated the classification accuracy and error rate to assess the performance of the proposed model. Therefore, the proposed RGG-Net model surpasses existing state-of-the-art methodologies with superior performance. The primary objective of the proposed approach is to enhance classification performance by effectively merging the benefits of ResNet-50 and GoogLeNet.

Tables 4 and 5 show the summary of the pre-trained models with and without freezing convolutional layers, along with the evaluation of performance metrics. Figure 4 show the experimental findings of accuracy analysis for the pre-trained CNN models with and without freezing the layers. Freezing the convolutional layers often leads to improved performance metrics because of the powerful generalization potential of the pre-trained data. The proposed approach provides better performance when fine-tuning is carried out on convolutional layers. Also, it is observed from Table 3 that DenseNet-201, MobileNetV1, and MobileNetV2 models produced better results than other pre-trained models. This analysis confirms that utilizing pre-trained models with frozen convolutional layers is an effective method for attaining high accuracy in certain applications, such as medical image interpretation.

Figure 6 shows the confusion matrix for freezing convolutional layers. The RGG-Net model outperforms other pre-trained models in classification accuracy and dependability, with the fewest False Positives and False Negatives. Models like MobileNetV2 and DenseNet-201 show low misclassification rates, while ResNet-101 and MobileNetV1 balance True Positives and True Negatives. VGG-19 Net, despite achieving True Positives, has a high incidence of False Positives, making it a reliable option. Figure 7 shows the without-freezing layers, which improves the models’ adaptability and accuracy, as evidenced by the confusion matrices. MobileNetV1 and GoogleNet perform well, however they have significantly higher False Negative rates. The proposed model (RGG-Net) is the most accurate and reliable classifier. Convolutional neural networks have been extremely effective in image classification, face recognition, and document analysis. However, due to rising efficiency and complexity, the DL model’s interpretability has deteriorated over time. Training a model with hundreds of layers and thousands of parameters to solve a problem like facial recognition makes the model hard to understand, debug, and trust. Convolutional Neural Networks (CNNs) are opaque systems that receive inputs and produce outputs with high accuracy but provide no insight into their internal workings. The Grad-CAM approach is a common visualization tool used to understand the training process of CNNs. It generates a heat map using target gradients, highlighting crucial areas of an image^58^. Figure 8 shows the Grad-CAM heatmaps, different colors represent varying levels of importance. Typically, warmer colors (reds and yellows) indicate higher importance, while cooler colors (blues and greens) indicate lower importance. In a healthy individual, the heatmap seems more consistent, suggesting that no specific feature of the spiral and wave image output is a prominent feature of the model. The Grad-CAM (Gradient-weighted Class Activation Mapping) technique offers visual representations of deep learning models by creating heatmaps that emphasize the specific areas of an image that have the greatest impact on the model’s predictions. Gradient calculation is to determine how much each feature map contributes to the prediction for a particular class $c$, calculate the gradient of the score for class $c$ with respect to the feature maps $A^k$ of the convolutional layer $l$. This gradient helps understand the sensitivity of the class score $y^c$ to the changes in the feature maps, as shown in Eq. (7),

7 $$\begin{aligned} \frac{\partial y^c}{\partial A^k} \end{aligned}$$

where $y^c$ is the score for class $c$, and $A^k$ is the activation map of the $k$-th filter in layer $l$.

Also, we compute the significance weights $\alpha ^k_c$ for each filter by taking the average of the gradients across all spatial dimensions of the feature map. This provides a quantification of the individual contribution of each filter to the class score:

8 $$\begin{aligned} \alpha ^k_c = \frac{1}{Z} \sum _{i,j} \frac{\partial y^c}{\partial A^k_{ij}} \end{aligned}$$

Here, $Z$ represents the number of spatial locations in the feature map, and $\frac{\partial y^c}{\partial A^k_{ij}}$ denotes the gradient at spatial location $(i,j)$. These weights indicate the importance of each filter’s activation in predicting class $c$. Finally, the weighted activation maps are combined to produce the Grad-CAM heatmap $L^c$. We utilize the computed weights $\alpha ^k_c$ to generate a weighted overall sum of the activation maps $A^k$, and employ the Rectified Linear Unit (ReLU) function to preserve only the positive contributions. The presented heatmap effectively identifies the specific areas within the image that exert the most impact on the class prediction.

9 $$\begin{aligned} L^c = \text {ReLU}\left( \sum _{k} \alpha ^k_c A^k\right) \end{aligned}$$

In Eq. (9), $\text {ReLU}$ (Rectified Linear Unit) ensures that only positive activations contribute to the heatmap, thus emphasizing the areas of interest in the image. In contrast, the heat map specifically identifies locations with more abnormalities for PD-affected individuals, suggesting that these regions are utilized to detect patterns associated with PD. Grad-CAM is a powerful tool for visually explaining and validating the prediction of the model. Clinicians can better understand the underlying motor impairments by detecting which parts of a hand drawing are the most indicative features of PD^58^.

### Comparative analysis

**Table 6:** Comparative analysis of related works with the proposed model based on various handwritten datasets for PD prediction.

Related works	Type of handwritten dataset	Dataset size	Models applied	Accuracy (%)
^5^	Parkinson’s disease handwriting database (PaHaW) (spiral images)	75	Ensemble classifier	74.76
^23^	Real-time dataset (word, syllable, or sentence)	75	SVM, Adaboost, KNN	81.3
^24^	NewHandPD dataset (spiral and meander images)	92	CNN	95.83
^27^	Not mentioned (handwriting dynamics)	66	SVM-RBF	83
^4^	Parkinson’s disease spiral drawings using digitized graphics tablet dataset (spiral images)	77	CNN	96.5
^59^	Publicly available UCI ML repository (spiral images)	80	Adaboost	96.02
^39^	Innovation and technology assessment of the Federal University (spiral and wave images)	204	DenseNet201 and VGG16	94% and 90%
^40^	Publicly available dataset (spiral and wave images)	469	VGG19-INC	98.45
^60^	PaHaW dataset (spiral images)	166	CC-Net	89.3
^61^	Publicly available dataset (spiral and wave images)	204	CNN	93.3
^62^	Publicly available dataset (spiral and wave images)	960	RF, KNN, SVM	83.1
^35^	NewHandPD dataset (circle, meander, and spiral images)	279	ResNet, VGG19, InceptionV3, and KNN	95
^63^	Publicly available UCI ML repository (spiral images)	77	Restricted Boltzmann machine (RBM) pipelined with multi-layer perceptron model classifier	95.32
^55^	K Scott Mader dataset (spiral and wave images)	3264	Modified Mobile Net V2	97.7
^64^	K Scott Mader dataset-augmented (spiral and wave images)	3264	EfficientNetB2	96.4
RGG-Net	K Scott Mader dataset-augmented (spiral and wave images)	3264	ResNet-50 and GoogLeNet	99.12

Table 6 compares various models based on handwritten datasets, size, model type, and accuracy. Shows that larger datasets generally lead to higher accuracy, and deep learning models (CNNs) often outperform traditional methods. The proposed RGG-Net model achieves the highest accuracy of 99.12% on the larger dataset. The findings of this study specify that the proposed model outperforms the existing ML and DL models in terms of accuracy in diagnosing Parkinson’s disease. The use of handwriting analysis to diagnose Parkinson’s disease shows great promise. DL models such as CNNs and variations have demonstrated and produced better performance in predicting Parkinson’s disease than other conventional ML algorithms. In most cases, model performance improves better with the increase in dataset size. This is observed by comparing models trained on different datasets, such as PaHaW and K Scott Mader, which are quite small. A more accurate and generalizable model may be possible with data augmentation using several image kinds (spiral, wave, meander, and circle). On the K Scott Mader dataset, the proposed RGG-Net model beats current state-of-the-art models, suggesting a potential breakthrough in handwriting analysis for PD diagnosis. Based on the findings from Table 6, it is demonstrated that handwritten datasets can be an effective tool for Parkinson’s disease prediction. Deep Learning models, especially CNN architectures, perform exceptionally well in classifying handwritten images when trained on larger datasets. Model performance can be improved by including varied handwriting samples, spirals, waves, and meanders. Significant improvements in early Parkinson’s disease prediction by handwriting analysis are possible with the help of the proposed RGG-Net model, which outperforms current approaches.

### Discussion

This work introduced several pre-trained models for PD prediction. The primary aim of this study in comparison to prior studies is to minimize losses and enhance the performance by utilizing a novel hybrid transfer learning fusion model (RGG-Net). Furthermore, using pre-trained models and the XAI technique enhances the reliability and clarity of the PD prediction model. Our proposed methodology has been found to significantly improve the performance of PD classification compared to the other methods. The recommended approaches and the corresponding metrics derived from each model are shown in Eqs. (1)–(6). Through the comparative analysis of various performance assessment metrics such as precision, recall, F1-score, and MCC, it can be inferred that our proposed approach improves classification performance and achieves higher accuracy.

The Grad-CAM heatmaps effectively highlight the differences between healthy control and PD-affected spiral and wave drawings. The model finds no significant disruptions for healthy individuals, leading to a more uniform heatmap. The model identifies and focuses on areas with tremors and inconsistencies for individuals with PD, as shown by the concentrated regions in the heatmap. This visualization helps understand how the model differentiates between healthy control and PD-affected drawings, offering valuable information for clinical applications. These highlighted areas represent the important features that influenced the outcomes. Table 6 compares the performance of the proposed model based on various handwritten datasets with previous studies. The limitation of this study is that the input dataset size is small. Our future work will explore the severity level of the disease through detailed feature engineering, custom loss functions, balancing techniques, and combining the pre-trained models using ensemble methods with different datasets to enhance the model’s performance. The dataset contains limitations such as limited size, dependency on data augmentation, poor resolution from resizing, and inadequate instances of Parkinson’s disease in various stages or severity levels, all of which make it difficult for the model to generalize and operate effectively in real-world settings. To expand the dataset, more real-life samples can be added, and advanced augmentation and preprocessing methods can be applied. In addition, people with different stages of PD severity and multimodal data can be combined. The intended enhancement can be made to the RGG-Net architecture for Parkinson’s disease (PD), which is enumerated below. Attention-based fusion or cross-attention methods allow the more focused, context-aware integration of information, thereby enhancing the identification of patterns in handwritten images. Grad-CAM must be accompanied by techniques like SHAP and LIME, which provide a more comprehensive understanding of the model’s decision-making and increase trust in healthcare applications. Combining handwriting data with other modalities, such as verbal and kinetic information, helps build a more complex model for diagnosing Parkinson’s disease. Using bigger pre-trained models, including EfficientNet or Vision Transformers (ViT), investigates the possibility of improved feature extraction and performance.

## Conclusion

We presented RGG-Net, a deep fusion model that combines ResNet-50 and GoogLeNet models for PD prediction using handwritten images. The proposed RGG Net model achieved a remarkable accuracy of 99.12%, surpassing ten previous pre-trained models. Adaptive feature fusion and hierarchical ensemble learning strengthen the hybrid model, which is vital for medical imaging applications. Also, the Grad-CAM technique is integrated with the proposed model for model interpretability and transparency in decision-making. To further highlight the efficacy of transfer learning in medical applications, our approach incorporates the assessment of ten additional pre-trained models. DenseNet-201, MobileNetV1, and MobileNetV2 models produced better results than other pre-trained models. The proposed RGG-Net model performs better than the other pre-trained models with and without freezing convolutional layers. These findings highlight the promise of pre-trained approaches for enhancing the precision and consistency of PD diagnosis. Moreover, the handwritten datasets can be considered the appropriate source for early PD prediction based on the results in Table 6. In summary, this study highlights the significant impact that deep learning performing better in medical diagnostics. It has the potential to greatly enhance the accuracy and speed of disease detection and intervention techniques.

This research will pave the way for further developments in the future. Firstly, the classification performance might be further improved by researching more pre-trained models and optimizing their integration. Furthermore, exploring larger and more varied datasets has the potential to enhance the model in terms of accuracy. Also, using sophisticated visualization techniques such as multi-modal data fusion and eXplainable AI technologies would strengthen the acceptability and confidence of clinical professionals by improving interpretability. Despite using pre-trained models, the problem of overfitting always exists, mostly in cases of a small dataset or an excessive number of parameters for the model. Finding the ideal equilibrium is still tricky, even if dropout or L2 regularization has possible advantages. Combining characteristics from many pre-trained models, such as ResNet-50 and GoogLeNet-using Hierarchical Ensemble Learning (HEL) and Adaptive Feature Fusion (AFF), complicates this procedure. The fusion technique must be fine-tuned for the most accurate feature representation for correct classification. The intricacy of the feature extraction procedure and the use of many deep learning models aggravate the computing load. It is always difficult to find strategies to improve the models so that inference takes less time without compromising accuracy. Creating precise predictions in real-time for use in healthcare settings is no small task. The research method could require some improvement to easily handle time-sensitive situations.

## Data Availability

The datasets used and/or analyzed during the current study are available in the Kaggle repository (https://www.kaggle.com/datasets/banilkumar20phd7071/handwritten-parkinsons-disease-augmented-data).

## Code availability

The code for cleaning and analysis used in this study is available at https://drive.google.com/drive/folders/1vIE3LuRopQoh69OHCSBvLQCjnaghNlsB?usp=sharing.
